# Community pharmacists’ response to complaints of gastroesophageal reflux: A simulated patient study in the Northern United Arab Emirates

**DOI:** 10.1371/journal.pone.0279922

**Published:** 2023-01-06

**Authors:** Fatima Boura, Moawia M. Al-Tabakha, Nageeb Hassan, Mohamad Darwich

**Affiliations:** 1 Department of Clinical Sciences, College of Pharmacy and Health Science, Ajman University, Ajman, United Arab Emirates; 2 Department of Pharmaceutical Sciences, College of Pharmacy and Health Science, Ajman University, Ajman, United Arab Emirates; University of Science and Technology of Fujairah, YEMEN

## Abstract

**Introduction:**

Patients frequently use gastric acid-reducing agents (ARAs) to treat symptoms affecting the gastrointestinal tract. Thus, the risk for drug–drug interactions (DDI) is a serious concern. This potentially makes the community pharmacist (CP) act as a primary intervention by providing the appropriate counseling and dispensing practice.

**Objective:**

To evaluate CPs’ counseling and dispensing practices regarding complaints of Gastroesophageal Reflux Disease (GERD), including recommending an appropriate course of action to prevent possible DDIs.

**Materials and methods:**

A simulated patient (SP) methodology was used in this study. The community pharmacies in Ajman and Sharjah were visited by SP who’s responsible for acting as a patient, and by an observer who’s responsible for focusing on the interaction between the SP and the CPs without engagement. Data were recorded using a preprepared data collection form. Performance feedback was sent to the CPs after concluding all visits. Counseling and dispensing scores were classified based on the total scores to poor, inadequate, and complete. Appropriateness of the pharmacist’s decision was defined as dispensing antacid and advising of separating doses apart in time.

**Results:**

A total of 150 community pharmacies was included in the data analysis. The findings of the current study demonstrated poor counseling and dispensing for the vast majority of the participants (81.3% and 67.3% of respondents, respectively). Only 4% of the CPs advised the SP to have a time interval between antacid and cefuroxime axetil. A significant difference in counseling scores was found between pharmacies located in Ajman and Sharjah (*p = 0*.*01*). Also, there was a significant difference in dispensing scores between independent and chain pharmacies (*p = 0*.*003*).

**Conclusions:**

The findings revealed inadequate counseling and dispensing practice by CPs. This study highlighted the need for continuous professional training programs to endow the CPs with the knowledge necessary for improving the CPs’ counseling and dispensing practices.

## Introduction

Millions of people worldwide suffer from digestive disorders, making it one of the major source of illness [1]. Gastroesophageal reflux disease (GERD) is one of the most common chronic digestive disorders in adults [1, 2]. It is a frequently presented disorder in the pharmacy [3]. According to the Montreal definition [4], GERD is a chronic disorder that occurs when stomach contents reflux, causing uncomfortable and recurrent symptoms and/or complications.

Although mortality is rare, GERD may have a significant economic impact that can adversely affect the healthcare systems and quality of life [5, 6]. According to a systematic review, the prevalence of GERD has risen in recent decades [1]. In the USA, GERD is a common symptom with a prevalence rate of 42% [7]. In North America, 20% of adults suffer from GERD symptoms weekly [5]. In Europe, digestive diseases caused more than 900,000 deaths in 2008 [8]. The estimated range of GERD prevalence in the Middle East was 8.7–33.1%, where most of the research came from Iran [9, 10]. Indeed, according to a study conducted in Saudi Arabia, the prevalence of GERD in Saudi Arabia is slightly greater than in Western countries and much greater than in East Asian countries [11]. Based on one study in Saudi Arabia, approximately 28.7% of adults suffer from GERD symptoms every week [11]. If this trend continues, more severe GERD complications could become more common, impacting patients’ quality of life and healthcare costs [12, 13].

Gastric acid-reducing agents (ARAs) are commonly used by patients in all fields of medicine to treat symptoms affecting the gastrointestinal tract GIT [5]. Because of the frequent use of ARAs, the risk of drug–drug interactions (DDIs) has become a growing concern [14]. Moreover, ARAs are widely available over the counter (OTC), which can increase the risk of DDIs, especially in patients using multiple medications concurrently without medical supervision [14, 15]. ARAs include antacids, histamine H2 receptor antagonists (H2RAs), and proton pump inhibitors (PPIs) [14]. These medications elevate gastric pH by different mechanisms [5, 16]. Consequently, this may affect the rate and/or extent of absorption of concurrently administered drugs with pH-dependent solubility, pH-dependent stability, or pH-sensitive release from a dosage form, and hence affect the activity of many other drugs [14].

Because of their wide distribution and accessibility, the community pharmacist (CP) has a great opportunity to act as a primary intervention. Several types of research were carried out to evaluate the practice of CPs. In this respect, using of simulated patient (SP) as an evaluation method to address problems in current pharmacy practices has received more attention in recent years [17]. The SP methodology has been explored as a rigorous and objective assessment tool [18]. A systematic review of the use of SP in pharmacy research has demonstrated the effectiveness of this approach in assessing the current practice compared to the traditional evaluation methods [19].

In United Arab Emirates (UAE) too, pharmacies are highly distributed and accessible. The number of pharmacies is more than 3500 in the UAE—a country of around 10 million—which is 1 pharmacy for every 2,857 people [20, 21]. As one of the most frequently presented complaints to the pharmacy is GERD [3, 22, 23], it is crucial to investigate the current practice of CP toward GERD in UAE to highlight the aspects that should be improved. Nevertheless, to our knowledge, there is no research exploring the current practice of CPs in response to GERD complaint originating from the UAE using an SP methodology [18]. Therefore, this research aimed (a) to evaluate CPs’ counseling and dispensing practices regarding complaints of GERD in the community pharmacy setting, (b) and to investigate if they can recommend an appropriate course of action to prevent possible DDIs.

## Materials and methods

### Study design

In this study, we used an SP methodology. An SP is an individual who has been trained to make a covert visit to a pharmacy to enact a pre-planned scenario that will assess a specific behavior in a natural environment provided by a pharmacy staff member while being indistinguishable from actual patients [24]. SP is also known as a pseudo patron, pseudo patient, standardized patient, pseudo customer, covert participant, shopper patient, disguised shopper, surrogate shopper, shopper patient, mystery shopper, or patient-actor [18].

### Study population and sampling method

Simple random sampling was applied to all community pharmacies in Ajman and Sharjah emirates that are registered with the Ministry of Health (MOH) using the statistical software IBM Statistical Package for Social Sciences (SPSS) Statistics for Windows, Version 26.0 (SPSS Inc., Chicago, Illinois). This sampling method was selected as the most appropriate data collection method for the current study.

### Sample size

For sample size calculation, we used the equation: N = (Za)^2[1-p]/ d^2. Za is standard normal variate, p is the prevalence of the condition which was obtained from an analogous study done in Saudi Arabia (16.3–74.6%) [25, 26], and d is the absolute error. According to this equation:, a sample of 135 pharmacies was needed [25] with the precision/absolute error of 7.5% and at type 1 error of 5%. However, 30% additional pharmacies were visited to increase the significance level of the findings. Thus, the final chosen sample size was 177.

### Research tool

#### Simulated patient

Community pharmacies in Ajman and Sharjah were visited by an SP (FB), 25 years old female master’s student from the College of Pharmacy at Ajman University, and by an observer (MD), a physician working in one of the hospitals in UAE. The visits commenced in November 2020 and lasted over three weeks. The SP (FB) acted as a patient and conducted the scenario. However, the observer (MD) was informed and trained to focus on the scenario without intervention.

#### Scenario

The scenario was developed after an extensive review of the published literature [18, 27, 28], guidelines about GERD treatment [29–31], and a physician and supervising faculty consultation. The selection of the most appropriate drug to cause DDI with ARAs for the SP scenario was in multiple steps:

1. We reviewed a recently published systematic review that aimed to comprehensively identify oral medications that have clinically meaningful DDIs with ARAs. Then, we listed all the drugs that have a clinically meaningful gastric pH-dependent mechanism of interaction.
2. The list was further reviewed to select the most appropriate drug for the SP scenario based on the following criteria:
  - **Criteria 1:** The drug should be suitable for the characteristics of the SP- a female in her twenties. This criterion was assessed by referral to the drug’s indication using Lexicomp (Lexicomp Inc., Hudson, Ohio) and UpToDate (UpToDate Inc., Waltham, Massachusetts). Common conditions were preferable. The final decision was made by a discussion with all research members.
  - **Criteria 2:** The drug should have a clinically meaningful interaction with all ARAs classes. This criterion was assessed by the same systematic review [14].
  - **Criteria 3:** The drug should not cause GERD-related symptoms as a side effect. This criterion was assessed by referral to Drug Information in Lexicomp (Lexicomp Inc., Hudson, Ohio).

According to the steps above, cefditoren pivoxil, cefpodoxime proxetil, cefuroxime axetil, and ketoconazole were the most suitable drugs for the scenario (S1 Table). Thus, we chose cefuroxime axetil as the drug that has DDI with ARAs because it is one of the top-dispensed antibiotics in the UAE [32].

Table 1 present the details of the standardized scenario. The SP was instructed not to provide any information unless requested. If the pharmacist failed to provide the desired information, the SP would then prompt the pharmacist by asking for the information. To ensure that only pharmacists were included in this study, the SP requested to speak to the pharmacist specifically. Visits were excluded if one of the following situations happened: the CP detected the SP visit, the SP and the observer failed to fill out specific element in the Data Collection Sheet (DCF), the SP did not follow the scenario, and the SP provided additional information that was not requested by the CP.

**Table 1:** The simulated patient scenario check.

	Scenario description
**Entry**	SP and the observer enter the pharmacy. SP asks: I need to talk to the pharmacist.
**Presenting symptoms**	SP asks: I need something to help with stomach acidity
**Pharmacist-patient interaction**	The CP is provided with the following information only upon questioning: • Who is the patient? The request is for herself. • What are the symptoms? Burning feelings tend to move upwards behind the breastbone and acid taste in the mouth. • How long have you had them? Three days • What treatment/s have you tried for these symptoms? Nothing. • Is any other medication taken? Only yesterday. She started taking cefuroxime axetil for tonsillitis after a physician’s visit. • Do you have any other medical conditions? only tonsillitis
**Additional information -if asked-**	• First time with this condition • Has yet to try any product before. • Has not seen a medical practitioner for the GERD condition. • Not breastfeeding/pregnant. • Not taking oral contraceptive • She is a non-smoker • She has important exams approaching, for which she is feeling anxious and stressed. • She has the usual food. She did not eat spicy foods
**Leaving and documentation**	After leaving a distance from the premise, the SP and the observer document all the information using DCF.

Abbreviations: SP = simulated patient, CP = community pharmacist, GERD = Gastroesophageal reflux disease, DCF = data collection form.

#### Data collection form

To reduce the possibility of biases associated with missing information, the SP (FB) and the observer (MD) recorded the data independently and immediately after they exited the pharmacy using a pre-designed DCF created by the researchers. Any discrepancies were resolved by discussion among themselves. Criteria were based on QuEST-SCHOLAR (stands for QUickly, Establish, Suggest, Talk, Symptoms, Characteristics, History, Onset, Location, Aggravating factors and Remitting factors), and WWHAM (stands for Who is it for?, What are the symptoms?, How long have the symptoms been present?, Any other medication being used at present?, and What Medication has been tried already?). Then modified to apply to pharmacy practice in UAE. The DCF consisted of four parts: 1. Details related to pharmacist and pharmacy include gender, city, type of pharmacy, location, time, and duration of the visit, 2. Counseling practice, 3. Dispensing practice, and 4. Appropriateness of treatment. The criteria were assessed using a dichotomous scale (yes/no).

To maintain the uniformity of the information recorded in the DCF in response to the pharmacist’s questions, the SP and the observer listed information related to the questions asked without providing any additional information spontaneously.

#### Training

A one-hour training session for the SP and the observer was carried out by a supervising faculty, one of the research team members, who had experience in this research methodology. They were trained for role-playing, and the DCF was to be filled after the visit. The SP has academic and research background and was involved in moderating SP sessions for educational tutorials. The performance was then evaluated by the researchers before the visits. In the evaluation process, the SP and the observer role-played the scenario in front of the research team with one of the researchers acting as a pharmacist. They were also trained to use non-jargon language.

#### Performance feedback

Performance feedback was sent to the CPs after the visits to the email address taken from the pharmacist incharge when verbal consent was obtained. The research team designed the performance feedback after referring to different resources [3, 33], and consists of six parts: 1. Processes of patient interaction (QuEST-SCHOLAR, and WWHAM), 2. Brief background about GERD, 3. What pharmacists need to know in a patient with GERD, 4. Management (non-pharmacological and pharmacological) 5. DDI with ARAs 6. When to refer to a doctor.

### Pilot study

Seven pharmacies were visited before the primary study to ensure the feasibility of the study, and those were excluded from the study analysis. No modifications were made to the scenario or the DCF, as the pilot study did not reveal any need for modification.

### Consent

Since it is essential to keep the identity of the SP hidden from CP because the CPs’ behavior would be affected if they suspected an SP visit, the authorized representative of the pharmacy (pharmacist in charge or manager) was contacted by the observer (MD) one month before the visit and informed him/her that an SP would visit their pharmacy in the following weeks. They were informed about the overall objectives of the study, and then verbal consent was obtained over the phone. The topic or the details of the scenario, the identity of the SP, and the time of the visit were not revealed to the participants. Also, the observer (MD) clarified to the enrolled CP that the study had a standardized feedback system to detect SP visits, where the CP has to call the observer on suspicions that an SP visit had taken place (the CPs were asked to provide the suspected visit’s date and the medicine dispensed). Based on the information provided, the researchers may decide whether the detected visit should be excluded. Participants were told that the collected data would be kept entirely confidential and anonymous. The observer (MD) gave the CPs his contact number so they can at any time refuse to participate or in case they want to report identifying the SP visit.

### Ethical approval

Ethical approval for conducting this study was obtained from the Institutional Research Ethics Committee at Ajman University (Ref number: P-H-F-2020-04-30).

### Outcomes

Data was collected on the adequacy of counseling and dispensing provided to the SP. Counseling and dispensing incidents were reported if any counseling and dispensing took place. The adequacy of counseling is defined as the number of questions that need to be asked (Total score = 9). The counseling elements include: who is the patient, what are the symptoms, how long have the symptoms been present, does the patient have any other medical conditions, is the patient taking any other medicines concurrently, has the patient tried any other treatments for GERD, any chance of pregnancy/breastfeeding, any history of allergies, and lifestyle and diet habits. Counseling score is divided into Poor (Total score = 0–2), Inadequate (Total score = 3–6), and Complete (Total score = 7–9). The adequacy of dispensing is defined as the number of dispensing elements provided to the patient after counseling and before the promptness (Total score = 7). The dispensing elements include: dose, how to take the medication, when to take the medication, duration of use, side effect, general DDIs, DDIs with cefuroxime axetil. Dispensing score is divided into Poor (Total score = 0–2), Inadequate (Total score = 3–5), and Complete (Total score = 6–7). Appropriateness of the treatment was defined as the appropriate course of action recommended by the CP to prevent possible DDIs with cefuroxime axetil. The correct outcome is to dispense antacid by advising the patient to take cefuroxime axetil 1 hour before or 2 hours after antacid and not to dispense H2RA or PPI [14].

### Statistical tests

Data were analyzed using the statistical software IBM SPSS Statistics for Windows, Version 26.0 (SPSS Inc., Chicago, Illinois). A 10% random check was conducted of all data entered. The p-value of *<0*.*05* was considered for statistically significant decisions. Shapiro-Wilk test, histogram, skewness, and kurtosis tests were done to examine the normality of the counseling score and dispensing score. Frequencies and percentages were used for discrete/categorical variables, while median and range were used to present continuous variables. Inferential statistics were used to determine and compare the counseling score, dispensing score, and appropriateness of the pharmacist’s decision across different pharmacist and pharmacy-related information using Pearson’s Chi-square test. In order to investigate the relationship between the counseling score, dispensing score, and appropriateness of the pharmacist’s decision across different pharmacists and pharmacy-related information, Spearman’s Rank Order Correlation (rho) was applied.

## Results

### Pharmacy and pharmacist-related information

Of the 177 included participants, 154 (87%) were approached because 23 CPs refused to participate in the study when contacted over the phone. One CP detected the SP visit on the spot, which was excluded from the analysis. Also, three SP visits were excluded from the analysis due to incomplete DCF. A total of 150 CPs were included in the data analysis. The participants include 62.7% males and 37.3% females. The majority of the participants were working in independent pharmacies (62.7%) located in Sharjah (74.7%). More than half of the visits lasted between 1:01 to 2:00 minutes (63.3%). The general pharmacy and pharmacist demographics of the study participants are shown in Table 2.

**Table 2:** Pharmacy and pharmacist-related information (n = 150).

Variables	Sub variables	Number (percentage) of pharmacists
**Gender**	**Male**	**94 (62.7)**
	**Female**	56 (37.3)
**City**	**Sharjah**	112 (74.7)
	**Ajman**	38 (25.3)
**Type of pharmacy**	**Independent**	94 (62.7)
	**Chain**	56 (37.3)
**Location**	**Shopping center**	9 (6)
	**Street**	119 (79.3)
	**Medical center**	22 (14.7)
**Visit Time**	**9:00–12:59**	27 (18)
	**13:00–16:59**	47 (31.3)
	**17:00–21:00**	76 (50.7)
**Duration**	**Less than & equal 1:00**	26 (17.3)
	**1:01–2:00**	95 (63.3)
	**2:01–3:00**	13 (8.7)
	**More than 3:00**	16 (10.7)

### Counseling practice of the pharmacist

**Fig 1:**
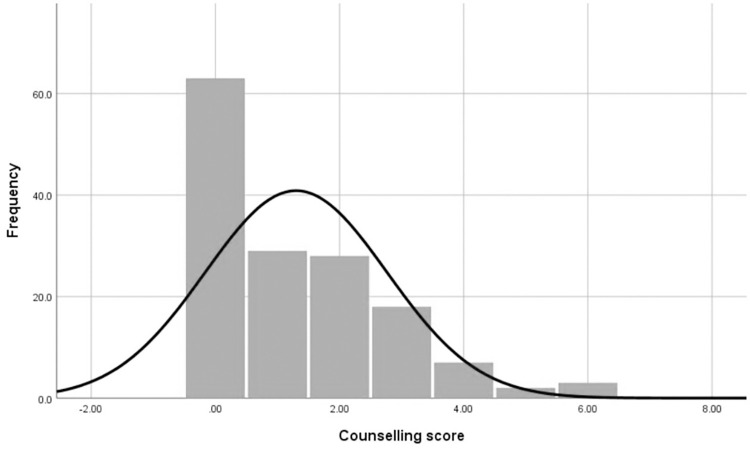
Distribution of counseling score.

Counseling scores ranged from the lowest score of zero for 63 (42.0%) participants to the highest score of six for 3 (2.0%) participants, as illustrated in Fig 1. The counseling scores were not normally distributed by referring to the Shapiro-Wilk normality test (*p* ≤ 0.05). The median score is 1.00 (interquartile range 0.00–2.00).

A poor counseling score was reported for the vast majority of the participants, 122 (81.3%). It was observed that most frequently asked questions were about the symptoms (34.7%) and the chance of pregnancy/breastfeeding (30.0%). Participants who asked about the medical condition, other medicines taken concurrently, and other treatments tried for GERD were 5 (3.3%), 6 (4.0%), and 7 (4.7%), respectively. Only two participants were asked about their lifestyle and diet habits (1.3%). Table 3 summarizes the counseling practice of the participants.

**Table 3:** Counseling practice across community pharmacists (n = 150).

Counseling inquiries	Number (percentage) of pharmacists asking the question	
	No	Yes
**Who is the patient?**	117 (78.0)	33 (22.0)
**What are the symptoms?**	98 (65.3)	52 (34.7)
**How long have the symptoms been present?**	107 (71.3)	43 (28.7)
**Does the patient have any other medical conditions?**	145 (96.7)	5 (3.3)
**Is the patient taking any other medicines concurrently?**	144 (96.0)	6 (4.0)
**Has the patient tried any other treatments for GERD?**	143 (95.3)	7 (4.7)
**Any chance of pregnancy/breastfeeding?**	105 (70.0)	45 (30.0)
**Any history of allergies?**	149 (99.3)	1 (0.7)
**Lifestyle and diet habits?**	148 (98.7)	2 (1.3)

Abbreviations: GERD = gastroesophageal reflux disease.

### Dispensing practice of the pharmacists

**Fig 2:**
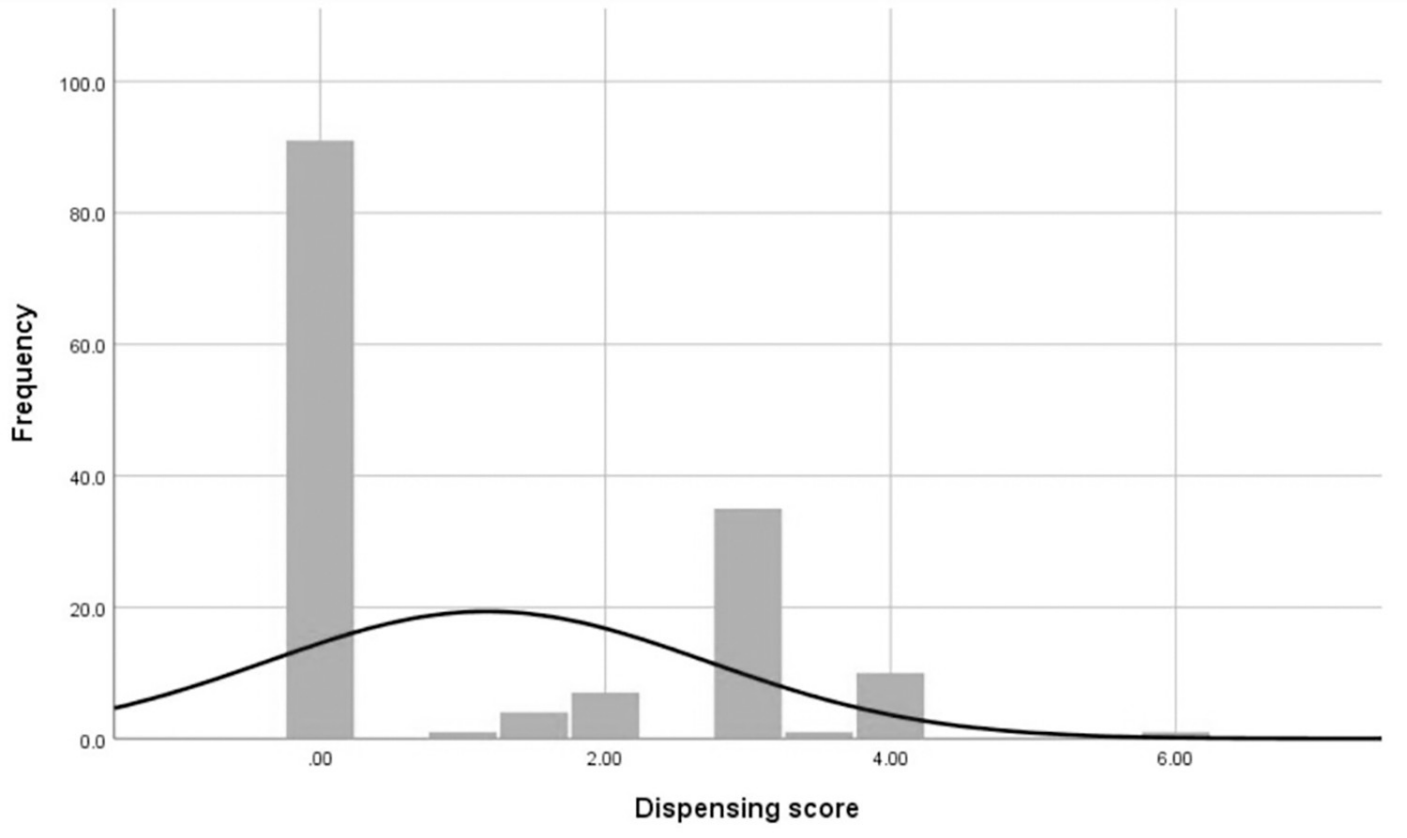
Distribution of dispensing score.

The dispensing score ranged from the lowest score of zero for 91 (60.7%) participants to the highest score of six 1 (0.7%) participant as shown in Fig 2. The dispensing scores were not normally distributed using the Shapiro-Wilk normality test (*P < 0*.*05*). The median score is 0.00 (inter-quartile range 0.00–3.00).

A poor dispensing score was reported for the vast majority of the participants, 101 (67.3%). The most given information by CPs, without SP promptness, in the majority of the treatment classes (PPI, Antacid, and Others) was dose information 50 (33.3%), 16 (10.7), and 1 (0.7%), respectively. None of the participants gave information about the DDIs with cefuroxime axetil. Details are tabulated in Table 4.

**Table 4:** Dispensing practice across community pharmacists (n = 150).

Dispensed drugs	Number (percentage) of pharmacists dispensing the medicine	Dispensing Statement						
		Dose	How	When	Duration	Side effect	General DDI	DDI with cefuroxime axetil
**PPI**	93 (63.0)	50 (33.3)	49 (32.7)	49 (32.7)	13 (8.7)	1 (0.7)	1 (0.7)	0 (0.0)
**H2RA**	1 (0.7)	0 (0.0)	0 (0.0)	0 (0.0)	0 (0.0)	0 (0.0)	0 (0.0)	0 (0.0)
**Antacid**	58 (38.7)	16 (10.7)	15 (10.0)	14 (9.3)	4 (2.6)	0 (0.0)	0 (0.0)	0 (0.0)
**Others**	7 (4.7)	1 (0.7)	1 (0.7)	1 (0.7)	1 (0.7)	0 (0.0)	0 (0.0)	0 (0.0)
**Lifestyle**	5 (3.3)							

Abbreviations: DDI = Drug-drug interactions, PPIs = proton pump inhibitors, H2RA = histamine H2 receptor antagonists

### Dispensed treatment

Only one participant suggested referral with treatment supply, while the rest suggested treatment without referral to a physician 149 (99.3%). The majority of the participants recommended PPI 93 (63.0%). Only one CP recommended H2RA (0.7%), and five (3.3%) participants advised about lifestyle modification. The details of the dispensed treatments are represented in Table 4.

More than three-quarters of the participants suggested single treatment 128 (85.3%). The most suggested single treatment was PPI 73 (48.6%). In comparison, the most dispensed combined treatment was PPI+Antacid 17 (11.3%). More details are presented in Table 5.

**Table 5:** Dispensed treatment.

Type	Number (percentage) of pharmacists dispensing the medicine	Therapeutic category	Number (percentage) of pharmacists dispensing the medicine	Drug Name		Number (percentage) of pharmacists dispensing the medicine
**Single treatment**	128 (85.3)	PPI	73 (48.6)	Omeprazole		40 (26.7)
				Esomeprazole		15 (10.0)
				Pantoprazole		14 (9.3)
				Lansoprazole		2(1.3)
				Rabeprazole		2 (1.3)
		Antacid	52 (34.7)	Antacid	(Alginic acid + Calcium Carbonate + Sodium Bicarbonate)	42 (28.0)
					(Aluminum Hydroxide + Magnesium Hydroxide)	7 (4.7)
					(Alginic acid + Calcium Carbonate + Magnesium Carbonate)	2 (1.3)
				(Antacid + Simethicone)	(Aluminum Hydroxide + Magnesium Hydroxide + Simethicone)	1 (0.7)
		Others	3 (2.0)	Simethicone		2 (1.3)
				Probiotic		1 (0.7)
**Combined treatment**	22 (14.7)	PPI + Antacid	17 (11.3)	PPI + Antacid	Omeprazole + (Alginic acid + Calcium Carbonate + Sodium Bicarbonate)	6 (4.0)
					Pantoprazole + (Aluminum Hydroxide + Magnesium Hydroxide)	3 (2.0)
					Pantoprazole + (Alginic acid + Calcium Carbonate + Sodium Bicarbonate)	4 (2.7)
					Esomeprazole + (Alginic acid + Calcium Carbonate + Sodium Bicarbonate)	1 (0.7)
					Omeprazole + (Aluminum Hydroxide + Magnesium Hydroxide)	1 (0.7)
				PPI + (Antacid + Simethicone)	Rabeprazole + (Aluminum Hydroxide + Magnesium Hydroxide + Simethicone)	1 (0.7)
					Omeprazole + (Aluminum Hydroxide + Magnesium Hydroxide + Simethicone)	1 (0.7)
		PPI + others	3 (2.0)	Pantoprazole + Probiotic		1 (0.7)
				Omeprazole + Licorice		1 (0.7)
				Omeprazole + Digestive enzymes		1 (0.7)
		H2RA + Antacid	1 (0.7)	Nizatidine + (Alginic acid + Calcium Carbonate + Sodium Bicarbonate)		1 (0.7)
		Antacid + others	1 (0.7)	(Alginic acid + Calcium Carbonate + Sodium Bicarbonate) + Dietary Supplement		1 (0.7)

Abbreviations: PPIs = proton pump inhibitors, H2RA = histamine H2 receptor antagonists

Only 6 (4.0%) CPs suggested having a time gap between the antacid and cefuroxime axetil. Nearly one-fifth of the participants think cefuroxime axetil is the reason for the SP GERD symptoms 32 (21%).

### Factors affecting pharmacist’s practice

There was a significant difference in counseling scores between pharmacies located in Sharjah and in Ajman (p-value 0.01). Moreover, there was a significant difference in dispensing scores between independent and chain pharmacies (p-value 0.003). However, there was no significant difference in the appropriateness of the pharmacist’s decision between the variables. The details are listed in Table 6.

**Table 6:** Factors affecting pharmacist’s practice.

Variables	Sub variables	Adequacy of counseling				Adequacy of dispensing				Appropriateness of treatment		
		Score			p-value*	Score			p-value*	Score		p-value*
		Poor	Inadequate	Complete		Poor	Inadequate	Complete		Yes	No	
**Gender**	**Male**	79	14	1	0.541	58	35	1	0.140	3	91	0.513
	**Female**	43	12	1		43	13	0		3	53	
**City**	**Sharjah**	96	16	0	**0.010**	75	37	0	0.212	4	108	0.646
	**Ajman**	26	10	2		26	11	1		2	36	
**Type of pharmacy**	**Independent**	76	17	1	0.893	54	39	1	**0.003**	3	91	0.513
	**Chain**	46	9	1		47	9	0		3	53	
**Location**	**Shopping center**	9	0	0	0.347	8	1	0	0.136	1	8	0.188
	**Street**	95	23	1		74	44	1		3	116	
	**Medical center**	18	3	1		19	3	0		2	20	
**Visit Time**	**9:00–12:59**	19	8	0	0.178	19	8	0	0.558	3	24	0.083
	**13:00–16:59**	42	5	0		33	13	1		2	45	
	**17:00–21:00**	61	13	2		49	27	0		1	75	
**Duration**	**Less than & equal 1:00**	22	3	1	0.575	17	9	0	0.070	0	26	0.456
	**1:01–2:00**	79	15	1		67	28	0		5	90	
	**2:01–3:00**	10	3	0		7	5	1		1	12	
	**More than 3:00**	11	5	0		10	6	0		0	16	

*Pearson’s Chi-square test

### Association

There was no relationship between the counseling and dispensing score (p-value 0.247), between the counseling score and appropriateness of the treatment (p-value 0.888), and between dispensing score and appropriateness of the treatment (p-value 0.083).

## Discussion

For the current and future of the pharmacy profession, there are some roles and responsibilities in practice that must be adequately addressed. Both dispensing and counseling play a comprehensive role to successful implement the appropriate treatment plan. They play a cornerstone in improving medication adherence for patients. Thus, decreasing hospitalization, and medical costs. This study, however, reveals poor counseling and dispensing practice among CPs in UAE. Similar findings were also found in UAE [34, 35]. The results of this study reflect that CPs need to be sufficiently prepared with the required skills to enable them to practice the profession properly in their pharmacies. Indeed, pharmacists must have sufficient skill because they are often the first source of knowledge of medicines for patients. A competent and well-trained pharmacist can deliver more successful health services and would definitely have a beneficial influence on health concerns. These findings should encourage the policymakers in the UAE of the significance of establishing pharmacists training programs for CPs on counseling and dispensing practice [36].

In the GCC countries, community pharmacy practice is changing, and pharmacists are expected to provide patient care with a public health focus [37]. In GERD, the pharmacist should establish the identity of any medication that has been tried to treat the symptoms or any other medication that has been taken [3]. Surprisingly, it was evident in our study that more than 95% of the pharmacist were unable to ask questions related to medical and medication history, such as: Does the patient have any other medical conditions? Has the patient tried any other treatments for GERD? Is the patient taking any other medicines concurrently? This finding was reasonably similar to the observation made in Saudi Arabia, where pharmacists didn’t show any concern regarding the use of concomitant drugs and history of drug allergy [38]. Although cefuroxime axetil was not the cause of GERD symptoms in our scenario, the patient medication history should be reviewed for drugs that may aggravate GERD. Comprehensive patient history is a cornerstone in evaluating a patient with GIT complaints. It is considered the first step to narrowing down the focus of the diagnostic and therapeutic plan for the patient. Thus, it may help the CP to prevent the suspected DDIs. These findings reinforce the critical need for a comprehensive approach to promoting counseling and dispensing practice of the CP in UAE. The Ministry of Health (MOH) should include aspects related to counseling and dispensing practice in Continuing Medical Education as a part of license requirements for pharmacists in the UAE. A cross-sectional study carried out in the US intended to evaluate the impact of educational programs on DDIs knowledge of the health care professionals (pharmacists, medical, and nursing students), revealed that significant improvement in healthcare professional students’ DDI knowledge was observed following participation in the educational session [39].

It is a serious concern to recognize that there is a significant lack of knowledge about the DDI with a common medical disorder such as GERD, which may negatively impact public health. Surprisingly, in our study, side effects and DDIs were the most commonly ignored types of information, in which this information was mentioned only by one CP. This result is much related to data observed by two studies in Qatar [28] and Ethiopia [40]. To make things even worse, a considerable number of pharmacists in our study who dispensed the medication was unable to detect or avoid DDI by correctly educating the SP on how to take cefuroxime axetil to reduce the effects of DDIs even though the included medications in this study are widely available in community pharmacies. Indeed, preventing drug interactions is an important goal to maximize patient benefit from medications [41]. As practice change has progressed, the individual’s need to learn has also changed. Internet access can facilitate the transfer of information, giving pharmacists an opportunity and motivation to learn, even if they do not have good education [42]. This will facilitate the usage of online drug interaction checker software to prevent possible DDI. Therefore, it is suggested to make internet access mandatory in all pharmacies in the UAE.

As with all studies, this study is not without limitations. First of all, the findings were restricted to only CPs working in Sharjah and Ajman. The outcomes would have been more significant if the study had been conducted in all UAE emirates. Secondly, the study sample was limited to two emirates of the UAE due to the COVID-19 lockdown and difficulties in transportation, even though the number of participating pharmacists was adequate to obtain a fair response rate. Thirdly, potential limitations relate to SP and observer fatigue from repetitive performances and dependence on SP memory recall. To prevent fatigue or changes in body language and acting performance, the researchers limited the number of simulated daily visits to ten. To reduce the risk of recall bias, we recruited an observer with the SP, and used a standardized DCF which was completed immediately after the visit. In addition, efforts were made to decrease the detection of the SP by the participants. For example, the observer accompanied the SP in each visit without engagement in the scenario, the observer was responsible for getting the verbal consent from the CP before the visits, and the verbal consent was obtained one month prior to the on-site visits. Fourthly, the SP and the observer were informed to resolve any discrepancies between them while filling the DCF out by discussion among themselves, which may bias the results. Fortunately, no discrepancies occurred. Fifthly, pharmacies were visited only once to minimize exposure during COVID-19. Consequently, circumstantial factors occurring at the time of the SP visit may have yet to capture the questioning skills of the pharmacist observed. Finally, due to the nature of the study, we could not report some demographics of the participants, like age and qualification. Thus, it was impossible to investigate the association between the demographic data of the CP and their counseling and dispensing practices. Despite these limitations, this study establishes a preliminary foundation for assessing pharmacist-patient interactions, particularly in the setting of mild diseases, and suggests some future research and training directions in this field.

## Recommendations

The results of our study emphasized the critical need for a minimum standard of practice and continuous professional education and training programs for CPs. The findings also suggest an urgent need for accessible scientific databases and electronic systems for all CPs to detect DDIs or other safety or efficacy measurements. More research is needed in this area to obtain more in-depth information on the counseling and dispensing practices of CPs in other emirates.

## Conclusion

This study explored the counseling and dispensing practice of CPs to manage symptoms of GERD in the UAE. The findings in this study revealed better dispensing practice than counseling practice by CPs. Both are, however, considered to be inadequate. Since too few questions were asked and provided by pharmacists, and only a few adopted structured approaches to questioning. Recommendations by the pharmacists were mostly inappropriate, and insufficient time was spent with the patient. This disappointing result could relate to many factors that need to be addressed to develop strategies to improve pharmacy practice.

## Supporting information

S1 Table Most suitable chosen drug for the simulated patient scenario. (DOCX)
