# Supplementary material for: Community pharmacists’ response to complaints of gastroesophageal reflux: A simulated patient study in the Northern United Arab Emirates
Source: PLoS One. 2023 Jan 6;18(1):e0279922. doi: 10.1371/journal.pone.0279922 (PMC9821703; doi:10.1371/journal.pone.0279922)
Supplement: S1 Table — (DOCX) [file pone.0279922.s001.docx]

S1 Table. Most suitable chosen drug for the simulated patient scenario.

| **Drug Name** | **Criteria checklist** | | | | | **Overall** |
| --- | --- | --- | --- | --- | --- | --- |
|  | **Criteria 1.** The drug is appropriate for the SP characteristic | | **Criteria 2.** The drug has clinically meaningful gastric pH-dependent DDI with all ARAs classes | | **Criteria 3.** The drug doesn’t cause GERD-related symptoms side effects |  |
|  | Decision | comment | Decision | comment |  |  |
| Acalabrutinib | No | Antineoplastic | Yes | | Yes | No |
| Atazanavir | No | Anti-HIV | Yes | | Yes | No |
| Bisacodyl DR | Yes | | No | No information available about interaction with H2RA and PPI | Yes | No |
| Bismuth subcitrate potassium, metronidazole, tetracycline hydrochloride | No | Used to treat H pylori. GERD-related symptoms may indicate failure of treatment and encourage pharmacists to refer the patient to the doctor | Yes | | Yes | No |
| Bosutinib | No | Antineoplastic | Yes | | Yes | No |
| Cefditoren pivoxil | Yes | Antibiotic | Yes | | Yes | Yes |
| Cefpodoxime proxetil | Yes | Antibiotic | Yes | | Yes | Yes |
| Cefuroxime axetil | Yes | Antibiotic | Yes | | Yes | Yes |
| Dabigatran etexilate mesylate | No | Anticoagulant | No | No interaction between H2RA and PPI | Yes | No |
| Dasatinib | No | Antineoplastic | Yes | | Yes | No |
| Delaviradine | No | Anti-HIV | Yes | | Yes | No |
| Digoxin | No | Antiarrhythmic | No | No information available about interaction with H2RA | Yes | No |
| Emtricitabine, rilpivirine hydrochloride, tenofovir disoproxil fumarate (Complera®) | No | Anti-HIV | Yes | | Yes | No |
| Erlotinib | No | Antineoplastic | Yes | | Yes | No |
| Ferrous sulfate | Yes | Iron salt | No | No information available about interaction with H2RA and PPI | No | No |
| Gefitinib | No | Antineoplastic | Yes | | Yes | No |
| Hyoscyamine | No | Anticholinergic | No | No information available about interaction with H2RA and PPI | No | No |
| Indinavir | No | Anti-HIV | No | No information available about interaction with Antacid | No | No |
| Itraconazole | Yes | Antifungal | Yes | | No | No |
| Ketoconazole | Yes | Antifungal | Yes | | Yes | Yes |
| Lapatinib | No | Antineoplastic | No | No interaction with all ARAs | Yes | No |
| Ledipasvir, sofosbuvir (Harvoni®) | No | Antihepaciviral | Yes | | Yes | No |
| Mefenamic acid | Yes | Analgesic | No | No information available about interaction with H2RA and PPI | No | No |
| Mesalamine | No | Used to treat Ulcerative colitis. GERD-related symptoms may indicate failure of treatment and encourage pharmacists to refer the patient to the doctor | No | No information available about interaction with H2RA and PPI | No | No |
| Nelfinavir | No | Anti-HIV | No | No information available about interaction with antacid | Yes | No |
| Neratinib | No | Antineoplastic | Yes | | No | No |
| Nilotinib | No | Antineoplastic | Yes | | No | No |
| Pazopanib | No | Antineoplastic | Yes | | Yes | No |
| Phenytoin | Yes | Anticonvulsant | No | No information available for the interaction between H2RA and PPI | Yes | No |
| Posaconazole oral suspension (Noxafil®) | Yes | Antifungal | No | No interaction between antacid and H2RA | Yes | No |
| Raltegravir (Isentress®) | No | Anti-HIV | No | No information available about interaction with H2RA | No | No |
| Riociguat | No | For pulmonary hypertension | No | No information available about interaction with H2RA | No | No |
| Risedronate sodium (Atelvia DR) | No | Bisphosphonate derivative  For osteoporosis | Yes | | No | No |
| Sofosbuvir, velpatasvir (Epclusa®) | No | antihepaciviral | Yes | | Yes | No |

Abbreviations: SP = simulated patient, DDI = Drug-drug interactions, ARAs= acid-reducing agents, GERD = Gastroesophageal reflux disease, H2RA = histamine H2 receptor antagonists, PPIs = proton pump inhibitors.
